# Cyclin D1 in well differentiated thyroid tumour of uncertain malignant potential

**DOI:** 10.1186/s13000-015-0262-8

**Published:** 2015-04-18

**Authors:** Monika Lamba Saini, Birgit Weynand, Jacques Rahier, Michel Mourad, Marc Hamoir, Etienne Marbaix

**Affiliations:** Anatomie pathologique, Cliniques universitaires Saint- Luc, Université catholique de Louvain, Avenue Hippocrate, 10/T-1, Brussels, 1200 Belgium; Anatomie pathologique, CHU Dinant-Godinne, Université catholique de Louvain, Avenue Docteur G. Thérasse, 1, Yvoir, 5530 Belgium; Service de transplantation rénale et chirurgie endocrinologique, Cliniques universitaires Saint-Luc, Université catholique de Louvain, B-1200 Bruxelles, Belgium; Service d’oto-rhino-laryngologie et chirurgie cervico-faciale, Cliniques universitaires Saint-Luc, Université catholique de Louvain, B-1200 Bruxelles, Belgium

**Keywords:** Thyroid, Papillary carcinoma, Follicular variant of papillary carcinoma, WDT-UMP, Follicular adenomatoid nodule

## Abstract

**Background:**

Encapsulated follicular tumours with equivocal papillary thyroid carcinoma (PTC) type nuclear features continue to remain a challenge despite the recent attempts to classify these borderline lesions. The term ‘well differentiated tumour of uncertain malignant potential (WDT-UMP)’ was introduced to classify these tumours. The present study aimed to evaluate the role of a cell cycle regulator like cyclin D1 in these tumours along with assessment of other well established PTC markers like galectin-3, HBME-1, CK19.

**Methods:**

Thirteen cases of metastatic PTC, papillary microcarcinoma and follicular variant of PTC (FVPTC) were identified from a histological review of 510 cases. In addition, 13 cases of a subset of follicular adenomatoid nodules with focal areas showing nuclear features characteristic of PTC, identified as WDT-UMP, were also analyzed. Immunohistochemical analysis of galectin-3, HBME-1, CK19 and the proliferation markers Ki67 and cyclin D1 was performed. Lesions were analyzed for cyclin D1 gene amplification by fluorescent in-situ hybridization.

**Results:**

All WDT-UMP lesions showed immunolabelling of cyclin D1, Ki67; 11/ 13 cases showed immunolabelling of CK19; 10/13 cases showed immunolabelling of HBME-1 and 4/13 cases showed immunolabelling of galectin-3. Surrounding benign adenomatoid areas showed no to faint focal staining in all thirteen cases of cyclin D1, HBME-1 and galectin-3. A low rate of cyclin D1 gene amplification was identified in a significant proportion of cells in the WDT-UMP lesions as compared to surrounding benign adenomatoid areas.

**Conclusions:**

Increased expression of cyclin D1 and amplification of its gene along with immunolabelling of HBME-1 in WDT-UMP lesions showing cytological features of papillary thyroid carcinoma within follicular adenomatoid nodules suggest that these areas could correspond to a precursor lesion of follicular variant of PTC. Overexpression of cyclin D1, associated with the amplification of the gene suggests that these WDT-UMP lesions are an intermediate between the benign and malignant groups making this group of lesions a reliable precursor of FVPTC.

**Virtual slides:**

The virtual slide(s) for this article can be found here: http://www.diagnosticpathology.diagnomx.eu/vs/1851820807142117

## Background

Papillary thyroid carcinoma (PTC) is the commonest endocrine malignancy. It constitutes 85-90% of all thyroid malignancies [1]. In USA and Belgium, PTC has shown the most rapid increase in incidence among all malignancies among women [1,2]. Among men, PTC is the malignancy with the second-fastest increase in incidence [3]. Despite the progress in understanding PTC, diagnostic difficulties and inter-observer variability is seen in encapsulated follicular lesions showing nuclear changes of PTC without definite invasion.

**Figure 1:**
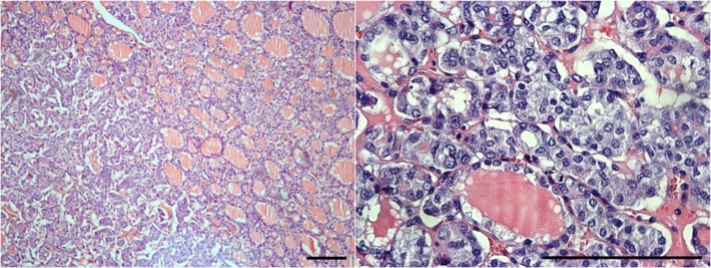
The WDT-UMP lesion, follicular variant of papillary carcinoma developing in a follicular adenomatoid nodule. Left panel, low power view with the WDT-UMP lesion at left; right panel, high magnification of the WDT-UMP lesion (H&E; bar = 200 μm).

Encapsulated follicular lesions, namely follicular adenoma/adenomatous nodule, follicular variant of PTC (FVPTC), and follicular thyroid carcinoma have always been the bane of the histopathologist [4]. Morphologically, PTC-nuclear changes such as nuclear clearing, nuclear grooves, enlargement and overlapping along with cytoplasmic pseudoinclusions are the diagnostic criteria of malignancy in a thyroid tumour irrespective of the growth pattern of the tumour or its aggressiveness [5]. However, when PTC-nuclear changes are ambiguous and there is no capsular/ blood vessel invasion in an encapsulated tumour, a new diagnostic term “well differentiated tumour of uncertain malignant potential (WDT-UMP)” has been introduced to describe this problematic entity [6], although there is no direct proof of its borderline malignant nature yet [7,8]. A review of cases of PTC led us to a particular diagnostic dilemma in a subset of follicular adenomatoid nodules with nuclear features partially characteristic of PTC (i.e., focal nuclear clearing, occasional nuclear grooves, nuclear enlargement, angulated nuclear contours and overlapping nuclei) but the histologic features did not display classic malignant characteristics (Figure 1). We decided to interrogate this controversial entity to determine its intermediate nature in progression to a malignant lesion.

In recent years, quite a few immunohistochemical markers have been implicated to identify PTC and also to distinguish it from benign mimics. The most promising and widely used ones are galectin-3, Hector Battifora Mesothelial cell (HBME)-1, and cytokeratin (CK) 19 [9]. Galectin-3 is involved in cell adhesion, growth and differentiation in multiple tissues [10]. CK19 shows a strong diffuse cytoplasmic immunoreactivity in PTC whereas HBME-1 is useful in the diagnosis of malignant tumours of follicular epithelial derivation [9,10]. PTCs show increased proliferation compared to normal thyroid tissue or benign lesions, when assessed with Ki67, a well characterized proliferation marker [11,12]. Cell cycle progression is brought about by cyclin-dependent kinases (CDKs) that are activated by cyclins including cyclin D1 and inactivated by CDK inhibitors. Cyclin D1 proto-oncogene regulates G1 to S-phase transition in cell cycle in different tissues [11,13]. Overexpression of cyclin D1, associated with the amplification of the *CCND1* gene, has been reported in oesophageal, lung, breast and head and neck carcinomas [14,15].

In this analysis, we used galectin-3, HBME-1, CK19, Ki67 and cyclin D1 to evaluate the borderline nature of this tumour. Identification of a biomarker that is consistently present in invasive as well as precursor lesion and absent in the adjacent normal thyroid tissue will provide evidence that this group of lesions is a reliable precursor of follicular variant of PTC (FVPTC).

## Methods

### Patients and selection of cases

Thirteen follicular adenomatoid nodules with focal areas showing PTC-nuclear changes were selected as WDT-UMP after a review of cases (1990 till date) done jointly by three authors (MLS, BW, and JR). The selection of the cases was made according to the WDT-UMP criteria described by Williams et al. [6]. The tumours were encapsulated with focal areas showing nuclear features of PTC like clearing, enlargement, grooves, angulated contours and overlapping (Figure 1). Papillary microcarcinoma and infiltrative metastatic PTC were selected as control groups according to the criteria of the World Health Organization [16]. FVPTC was selected by the criteria described by LiVolsi et al. [17]. Since only thirteen cases of the WDT-UMP lesion were available, we selected thirteen cases of several categories of PTC, including papillary microcarcinoma, FVPTC and metastatic PTC, and compared them to the thirteen cases of WDT-UMP lesion. The study was approved by the Ethics Committee of the Université catholique de Louvain.

### Immunohistochemistry

Formalin-fixed paraffin-embedded 5-μm-thick sections were deparaffinised and rehydrated. Endogenous peroxidase activity was blocked with 3% hydrogen peroxide. Antigen retrieval was performed in 0.01 M citrate buffer (pH 5.8) inside a boiling water bath for 75 min followed by incubation in 0.05% Triton X-100, 0.05 M Tris–HCl, pH 7.4 containing 10% goat serum to block non-specific binding. Primary antibody incubation (Table 1) was carried out overnight at room temperature. Slides were incubated with DAKO EnVision + System™, HRP for 60 minutes and the reaction visualized using 3, 3′-diaminobenzidine tetrahydrochloride (DAB). Controls were incubated with 0.05 M Tris–HCl, pH 7.4 containing 1% goat serum in place of the primary antibody, and no staining was observed. The adjacent normal thyroid tissue was used as an internal control for immunolabelling.

**Table 1:** **Clones of the antibodies used in the study**

**Antibody**	**Clone**	**Dilution**	**Manufacturing company**
**Galectin-3**	9C4	1:200	Novocastra, Newcastle, UK
**HBME-1**	HBME-1	1:50	DakoCytomation, Glostrup, Denmark
**CK19**	b-170	1:200	Novocastra, Newcastle, UK
**Cyclin D1**	SP 4	1:25	Thermo Scientific, Fremont, CA, USA
**Ki67**	MIB 1	1:100	DakoCytomation, Glostrup, Denmark

### Evaluation of immunostaining

Digitalization of the scanned slides was done at a 20x magnification by SCN400 slide scanner (Leica, Wetzlar, Germany). Scanned slides were analyzed using Tissue IA (Leica Biosystems, Dublin, Ireland). The tumour tissue was delineated manually and folds, bubbles and dirties were excluded from the analysis. Colour deconvolution was applied using hematoxylin and DAB matrices of the software. Nuclear algorithms were applied for cyclin D1 and Ki67 immunostaining, keeping the parameters constant for all slides. Thresholds were adjusted for tissue and DAB detection and also for nuclear segmentation. Percentage of nuclei with positive staining and corresponding staining intensity were generated. Cyclin D1 immunoreactivity was evaluated as: negative; grade 1, focal staining in less than 25% of tumour cells; grade 2, staining in 25–50% of tumour cells; and grade 3, diffuse staining in more than 50% of tumour cells [18]. Ki67 staining was scored according to percentage of cells showing nuclear staining, as less than 1%, 1 to 5%, and 5 to 20% [18].

For CK19 and HBME-1, a distinct staining in cytoplasm and cell membranes was considered as significant. Galectin-3 showed nuclear and cytoplasmic staining in thyroid carcinomas. The staining in the normal thyroid tissue surrounding the follicular adenomatoid nodules was also evaluated at the same time as the tumour.

### Fluorescent in-situ hybridization

Testing for cyclin D1 gene amplification was done using the Vysis CCND1/CEP 11 kit (Abbott SA, Louvain-la-Neuve, Belgium), including probes for cyclin D1 gene (11q13, spectrum orange) and chromosome 11 (11p11.11q11, spectrum green). Briefly, the 4 μm-thick sections were deparaffinised, rehydrated and placed in a HCl bath (0.2 N, 10 min, room temperature). Slides were immersed in 0.01 M citrate buffer (pH 6.1) inside a water bath (20 min, 100°C) and then treated with Tissue digest (Insitus Biotechnologies, Albuquerque, NM, USA) for 85 minutes at 38°C to unmask target DNA sequences followed by sequential washing with deionised water and 2X SSC solution. Probes (10 μl) were added to each slide and denaturation was performed by placing the slides on a hot plate (90°C, 13 min). Hybridization was carried out in a prewarmed humidified box kept in a 37°C incubator for 16 hours. Post-hybridization wash was done and nuclei were counterstained with 4′, 6-diamidino-2-phenylindole and signals from 200 nuclei were counted under a fluorescent microscope (Zeiss, Axioplan 2).

### Statistical analysis

Computations were performed using GraphPad Prism version 5.04 for Windows [19]. One-way ANOVA test was employed to look for significant difference in the immunolabelling of Ki67 and cyclin D1. Tukey’s post hoc test was done in cases of significance. Paired *t*-test was done to determine a difference in cyclin D1 gene amplification by FISH in different PTC variants and the surrounding normal thyroid tissue or benign adenomatoid areas. Galectin-3, HBME-1 and CK19 staining in the adenomatoid and neoplastic areas were analysed with a chi-square test. Statistical significance was defined at the level of p < 0.05 (two-tailed test).

## Results

The clinical details of the WDT-UMP cases are summarised in Table 2. Eleven out of thirteen patients were females, effectively drawing a male:female ratio of 1:5.5. The average age at presentation was 37 ± 18 (mean ± SD) years, the oldest being 63 years and the youngest being 13 years. Twelve out of 13 patients were available for follow-up for a period ranging from 36 to 132 months (average 74 months). None of them showed recurrence of the disease. Focal areas of WDT–UMP areas inside the follicular adenomatous nodule ranged in size from 3 mm to 1 cm. The WDT-UMP areas had thyroid follicles lined by cuboidal epithelium and showed relatively less colloid compared to the surrounding adenomatous areas. The nuclei were enlarged 2 to 4 times compared to that of the normal thyroid follicular cells and showed clearing, angulated contours, grooves and occasional nuclear overlapping and cytoplasmic pseudoinclusions.

**Table 2:** **General findings and follow up of the WDT-UMP lesion group**

**S.No**	**Age at presentation**	**Sex**	**Presenting complaints**	**Diagnosis since**	**Prognosis**
1	63 years	M	Nodular goitre	10 years	No recurrence
2	56 years	F	Nodular goitre	5 years	No recurrence
3	55 years	F	Nodular goitre	5 years	No recurrence
4	57 years	F	Nodular goitre	10 years	No recurrence
5	50 years	F	Nodule with dysphonia	3 years	No recurrence
6	50 years	F	Multinodular goitre	13 years	No recurrence
7	39 years	F	Multinodular goitre	3 years	No recurrence
8	37 years	M	Mutinodular goitre	5 years	No recurrence
9	36 years	F	Nodule with hepatitis C	9 years	No recurrence
10	34 years	F	Multinodular goitre	13 years	Lost to follow up
11	27 years	F	Nodular goitre	11 years	No recurrence
12	17 years	F	Nodular goitre	7 years	No recurrence
13	13 years	F	Nodular goitre	6 years	No recurrence

### Immunohistochemical results

**Figure 2:**
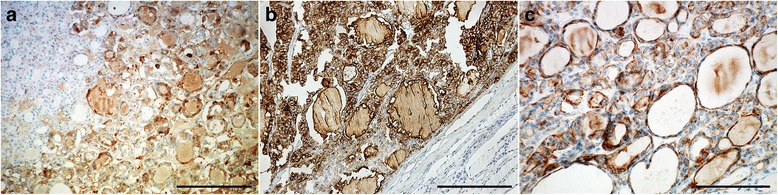
Immunolabelling of the WDT-UMP lesion for galectin-3 **(2a)**, HBME-1 **(2b)**, and CK19 **(2c)** with the corresponding adenomatoid nodule (bar = 200 μm).

All cases of metastatic PTC showed immunolabelling for HBME-1, CK19 and galectin-3. Galectin-3 was expressed in FVPTC (61%), papillary microcarcinomas (69%), and only in 30% cases of the WDT-UMP (Figure 2a). It showed no immunostaining in normal and adenomatoid areas. Membranous staining of HBME-1 was seen in FVPTC (76%), papillary microcarcinomas (92%) and WDT-UMP (76%) (Figure 2b). No immunostaining was found in the adjacent normal thyroid tissue. A non specific weak reactivity for HBME-1 was present at the luminal surface of some follicular cells in adenomatoid areas. CK19 showed diffuse cytoplasmic staining in FVPTC (84%), papillary microcarcinomas (84%), and WDT-UMP (84%) (Figure 2c, Table 3). The normal areas were unreactive for CK19; however, 80% of benign adenomatoid areas showed focal weak staining for CK19. The chi-square test showed significant difference of galectin-3 and HBME-1 staining between WDT-UMP and the surrounding adenomatoid areas (p < 0.05).

**Table 3:** **Galectin-3, HBME-1 and CK19 immunolabelling in the variants of PTC**

**Variant**	**FVPTC**	**WDT-UMP lesion**	**Papillary microCa**	**Metastatic papillary Ca**
**Galectin-3**	08/13	04/13	09/13	13/13
**HBME-1**	10/13	10/13	12/13	13/13
**CK19**	11/13	11/13	11/13	13/13

**Figure 3:**
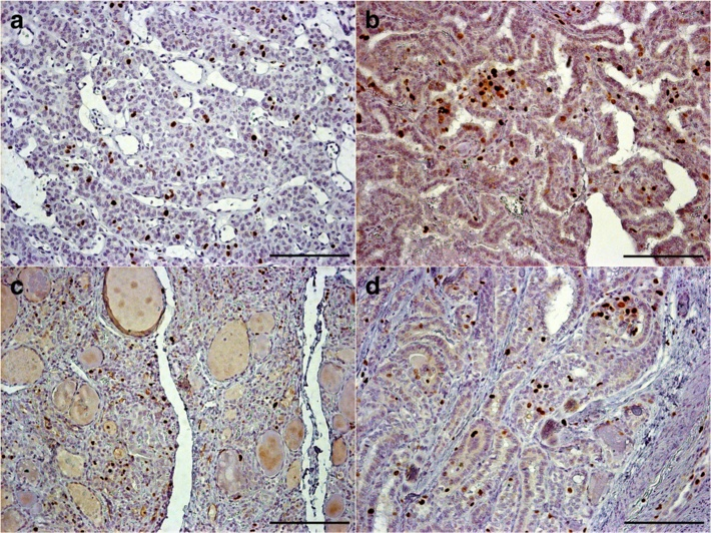
Ki67 immunolabelling in the WDT-UMP lesion, FVPC in FA **(3a)**, papillary microcarcinoma **(3b)**, FVPC **(3c)** and metastatic PTC **(3d)** (bar = 200 μm).

Proliferation as assessed by Ki67 staining revealed low scores in these slow growing lesions (Table 4, Figure 3). Ki67 was not detected in normal thyroid tissue, which also served as a control. A few cells were labelled in the benign follicular adenomas but the proportion of immunolabelled cells increased in the PTC variants. A one way ANOVA test showed significant difference between all the lesions (p < 0.01). Post hoc comparisons indicated that the mean score for the metastatic PTC was significantly different from benign follicular adenomatoid areas, WDT-UMP and papillary microcarcinoma at p < 0.01.

**Table 4:** **Ki67 immunolabelling in the variants of PTC**

**Variants of PTC**	**Percentage of Ki67 immunolabelled tumour cells**			
	**No staining**	**Grade 1 (<1%)**	**Grade 2 (1–5%)**	**Grade 3 (6-20%)**
**FVPTC (n = 13)**	**1**	**3**	**3**	**6**
**WDT-UMP lesion (n = 13)**	**0**	**2**	**6**	**5**
**Papillary microCa (n = 13)**	**2**	**3**	**6**	**2**
**Metastatic papillary Ca (n = 13)**	**0**	**0**	**2**	**11**

**Figure 4:**
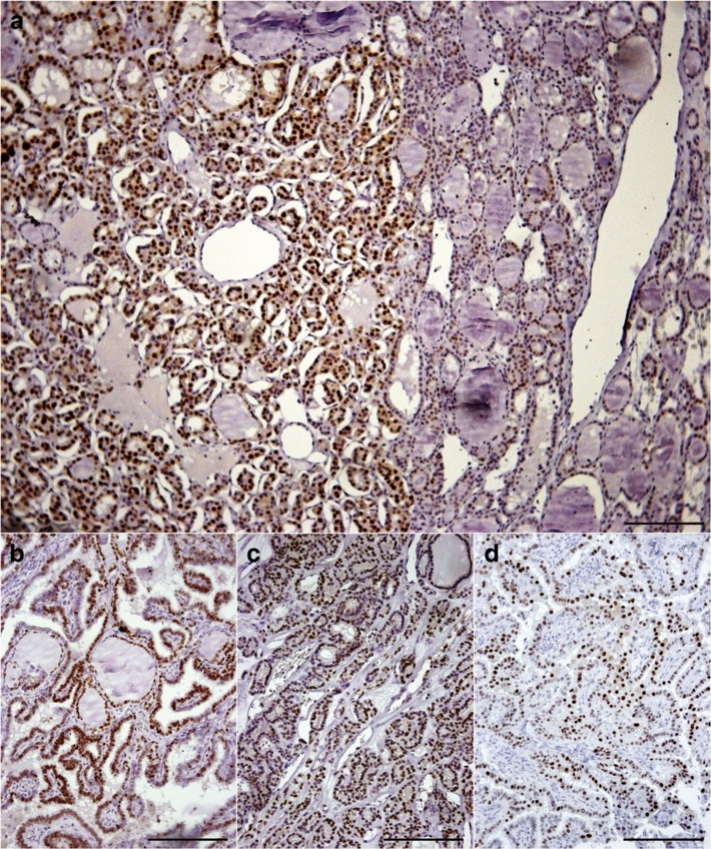
Cyclin D1 immunolabelling in the WDT-UMP lesion, FVPC in FA **(4a)**, papillary microcarcinoma **(4b)**, FVPC **(4c)** and metastatic PTC **(4d)** (bar = 200 μm).

Seven cases (54%) of WDT-UMP showed intense grade 3 cyclin D1 nuclear staining; three cases (23%) showed grade 2 staining and three cases (23%) showed grade 1 focal nuclear staining. The corresponding adenomatoid areas depict absent to faint focal nuclear staining (Figure 4a). Twelve out of 13 FVPTC showed nuclear staining of cyclin D1 with 6 (43%) showing grade 3 diffuse staining. (Figure 4c, Table 5). Eleven out of 13 papillary microcarcinomas showed nuclear staining of cyclin D1 (Figure 4b) with grade 3 diffuse nuclear staining seen in 5 (38%) of the tumours. All the metastatic carcinomas showed nuclear cyclin D1 immunolabelling with 69% depicting intense, grade 3 diffuse nuclear staining (Figure 4d). No immunolabelling was seen in normal thyroid tissue. There was no significant difference in the immunolabelling between the different variants according to ANOVA test. However, cyclin D1 immunolabelling in the WDT-UMP was significantly different from the surrounding benign adenomatoid areas (p = 0.012, using paired *t* test).

**Table 5:** **Cyclin D1 immunolabelling in the variants of PTC**

**Variants of PTC**	**Percentage of cyclin D1 immunolabelled tumour cells**			
	**No staining**	**Grade 1 (<25%)**	**Grade 2 (25-50%)**	**Grade 3 (>50%)**
**FVPTC (n = 13)**	**1**	**4**	**2**	**6**
**WDT-UMP lesion (n = 13)**	**0**	**3**	**3**	**7**
**Papillary microCa (n = 13)**	**2**	**4**	**2**	**5**
**Metastatic papillary Ca (n = 13)**	**0**	**2**	**2**	**9**

### FISH results

**Figure 5:**
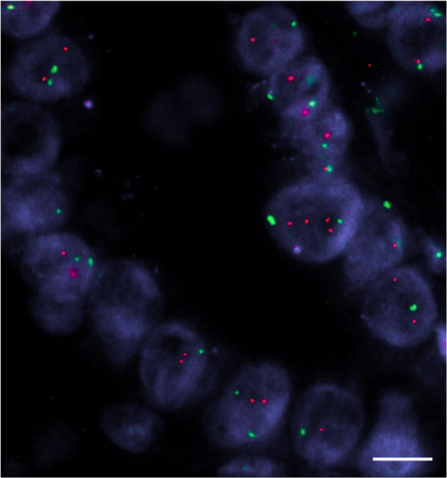
Amplification of cyclin D1 gene seen in the WDT-UMP lesion, FVPC in FA using FISH. Cyclin D1 gene appears in red whereas chromosome 11 centromeric repeat region is green (bar = 20 μm).

A low rate of cyclin D1 gene amplification was found in all the variants of PTC, metastatic PTC (19 ± 8% of cells), FVPTC (13 ± 4%), papillary microcarcinoma (12 ± 6%) and WDT-UMP (12 ± 5%). Ratio of CCND 1/CEP11 amplification ranged from 1.5 to 2.5 (Figure 5). Cyclin D1 amplification was significantly higher in each PTC variant than in normal tissue (p < 0.001) and in WDT-UMP than in surrounding adenomatoid areas (p = 0.002).

## Discussion

Immunohistochemical studies on thyroid borderline lesions such as WDT-UMP have always focused on the panel of well recognised markers like galectin-3, HBME-1 and CK19 [5,20]. The recognition of WDT-UMP using cyclin D1 as a marker is a first as per the available English literature. We found significant cyclin D1 amplification and intense nuclear cyclin D1 immunostaining in the WDT-UMP lesion as compared to normal tissue and adenomatoid areas. Increased proliferation was also detected by Ki67 immunostaining.

Most cases of PTC variants and the WDT-UMP lesion showed immunolabelling of HBME-1. Galectin-3 was immunolabelled in only 30% of the WDT-UMP lesion compared to 70-100% of PTC variants. CK19 was immunolabelled in most cases of PTC variants and in the WDT-UMP lesion but a weak immunostaining was also detected in the surrounding benign adenomatoid areas. Several investigators have reported CK19 immunostaining of benign thyroid lesions as observed in the present study [9,20]. Nasr et al. [20] have proposed that a lack of CK19 immunolabelling completely rules out PTC.

The idea of tumorigenesis in thyroid beginning from chromatin clearing with nuclear atypia, followed by appearance of nuclear grooves and pseudoinclusions, and then developing into a FVPTC running the entire gamut of changes from non-neoplastic disease to follicular adenoma and then to carcinoma has been suggested by Pennelli et al. [21]. We propose that follicular lesions with equivocal nuclear features are indeed a precursor of FVPTC wherein a progressive transformation to a malignant phenotype takes place as determined by cyclin D1. Cyclin D1 regulates G1 to S phase progression and its overexpression has been reported in various carcinomas [14,15,22]. Cyclin D1 was identified as a strong candidate diagnostic marker for PTC and its variants, particularly the WDT-UMP lesion. No statistical difference was observed between WDT-UMP and other variants of PTC for the frequency of cyclin D1 immunolabelling. However, cyclin D1 immunolabelling in the WDT-UMP was significantly different from the surrounding benign adenomatoid areas. HBME-1 and galectin-3 were not labelled in the normal thyroid tissue or the benign adenomatoid areas but were labelled in the WDT-UMP lesions, strongly supporting the hypothesis. However, neither of the cases in our study had invasion or cervical lymphadenopathy at surgery, nor recurrence at follow-up. Other studies have also reported a rather uneventful follow-up of WDT-UMP lesions [23].

## Conclusions

Increased expression of cyclin D1 and amplification of its gene along with immunolabelling of HBME-1 in WDT-UMP areas showing cytological features of PTC within follicular adenomatoid nodules suggest that these areas could correspond to a precursor lesion of follicular variant of papillary thyroid carcinoma. Since there is a significant overexpression of cyclin D1 in PTC and its variants, we also advocate that antibodies against cyclin D1 should be included in the immunohistochemical panel as a diagnostic marker for PTC.

### Take home message

- Cyclin D1 was identified as a strong candidate diagnostic marker for PTC and its variants, including papillary microcarcinomas and also the WDT-UMP lesion. The recognition of WDT-UMP using cyclin D1 as a marker is a first as per the available English literature.
- HBME-1 was immunolabelled at the membrane in most cases of the PTC variants and also in the WDT-UMP lesion but not in the normal thyroid tissue or the benign adenomatoid areas.
- Identification of a biomarker that is consistently present in invasive as well as the WDT-UMP lesion and absent in the adjacent normal thyroid tissue provides evidence that this group of lesions is a reliable precursor of FVPTC. Increased expression of cyclin D1 and amplification of its gene along with immunolabelling of HBME-1 in WDT-UMP areas showing cytological features of papillary thyroid carcinoma within follicular adenomatoid nodules suggest that these areas could correspond to a precursor lesion of follicular variant of papillary thyroid carcinoma.
